# The genome sequence of the Loggerhead sea turtle, *Caretta caretta* Linnaeus 1758

**DOI:** 10.12688/f1000research.131283.2

**Published:** 2023-06-27

**Authors:** Glenn Chang, Samantha Jones, Sreeja Leelakumari, Jahanshah Ashkani, Luka Culibrk, Kieran O'Neill, Kane Tse, Dean Cheng, Eric Chuah, Helen McDonald, Heather Kirk, Pawan Pandoh, Sauro Pari, Valeria Angelini, Christopher Kyle, Giorgio Bertorelle, Yongjun Zhao, Andrew Mungall, Richard Moore, Sibelle Vilaça, Steven Jones

**Affiliations:** 1 Genome Science and Technology Graduate Program, University of British Columbia, Vancouver, British Columbia, V6T 1Z4, Canada; 2 Canada's Michael Smith Genome Sciences Centre, Vancouver, British Columbia, V5Z 4S6, Canada; 3 Fondazione Cetacea Onlus, Riccione, RN, 47838, Italy; 4 Forensic Science Department, Trent University, Peterborough, Ontario, K9L 0G2, Canada; 5 Environmental and Life Sciences Graduate Program, Trent University, Peterborough, Ontario, K9L 0G2, Canada; 6 Department of Life Sciences and Biotechnology, University of Ferrara, Ferrara, FE, 44121, Italy; 7 Department of Medical Genetics, University of British Columbia, Vancouver, British Columbia, V6T 1Z4, Canada

**Keywords:** Caretta caretta, Loggerhead sea turtle, genome sequence, chromosomal, reptile

## Abstract

We present a genome assembly of *Caretta caretta* (the Loggerhead sea turtle; Chordata, Testudines, Cheloniidae), generated from genomic data from two unrelated females. The genome sequence is 2.13 gigabases in size. The assembly has a busco completion score of 96.1% and N50 of 130.95 Mb. The majority of the assembly is scaffolded into 28 chromosomal representations with a remaining 2% of the assembly being excluded from these.

## Species taxonomy

Eukaryota; Metazoa; Chordata; Craniata; Vertebrata; Euteleostomi; Archelosauria; Testudinata; Testudines; Cryptodira; Durocryptodira; Americhelydia; Chelonioidea; Cheloniidae; Caretta; *Caretta caretta* Linnaeus 1758 (NCBI txid 8467).

## Introduction

The loggerhead sea turtle, *Caretta caretta*, is one of only seven extant marine turtle species and is globally distributed throughout the subtropical and temperate regions of the Mediterranean Sea and Pacific, Indian and Atlantic Oceans ( Wallace *et al.,* 2010, Casale and Tucker, 2015). The species is divided in various Regional Management Units (RMUs) and management units (MUs) that vary greatly by population size, geographic range, and population trends ( Wallace *et al.,* 2010, Casale and Tucker, 2015, Shamblin *et al.*, 2014). Events such as fisheries bycatch ( Caracappa *et al.,* 2018, Pulcinella *et al.,* 2019), human intrusion and disturbance ( Mazaris *et al.,* 2009), oceanic pollution ( Savoca *et al.,* 2018), and climate change and severe weather ( Alduina *et al.,* 2020) have caused the global population to continuously decline ( Casale and Tucker, 2015). Consequently, the highly migratory *C. caretta* requires the collaborative efforts of numerous international conservation and protection organizations ( Species at Risk Act, 2002), and is currently listed as Vulnerable by the International Union for the Conservation of Nature (IUCN) ( Casale and Tucker, 2015). The genome of *C. caretta* was sequenced as part of the Canadian BioGenome Project (CBP) and CanSeq150 initiatives. The *C. caretta* genome will provide insights into genomic diversity and architecture, and inform conservation genomics applications.

## Methods

### Sample collection

Blood samples from an adult female and a juvenile of unknown sex were collected from the Fondazione Cetacea (43.9940 N, 12.6745 E) by Nicola Ridolfi (veterinarian; Fondazione Cetacea). Animal husbandry and welfare were overseen by Fondazione Cetacea. The specimens were transferred to Canada with two CITES permits between institutions (IT002 and CA027).

### Sample extraction, library construction and sequencing

High-molecular weight (HMW) DNA was extracted from nucleated blood using the MagAttract HMW DNA kit (QIAGEN, Germantown, MD, USA). Nanopore genome libraries were constructed according to manufacturer instructions and sequenced using the PromethION instrument (Oxford Nanopore Technologies). A PCR-free genome library was sequenced in a multiplexed pool of an Illumina NovaSeq 6000 instrument S4 flowcell with paired-end 150 bp (PE150) reads. A Hi-C library was constructed using the Arima-HiC kit 2.0 (Arima Genomics, San Diego, CA) and the Swift Biosciences Accel-NGS 2S Plus DNA Library Kit (Integrated DNA Technologies, Mississauga, ON, Canada) and subjected to PE150 sequencing on an Illumina NovaSeq 6000 instrument. All lab work were performed at Canada’s Michael Smith Genome Sciences Centre at BC Cancer.

### Genome assembly

Assembly was carried out using Redbean ( Ruan and Li, 2019), followed by four rounds of racon ( Vaser *et al.,* 2017) polishing and medaka (medaka, n.d.) polishing. Scaffolding with Hi-C data was carried out using nf-core/hic workflow ( Servant and Peltzer, 2019), Salsa ( Ghurye *et al.,* 2019) and LongStitch ( Coombe *et al.,* 2021). The Hi-C scaffolded assembly was polished using Illumina short-reads using Pilon ( Walker *et al.,* 2014). Four rounds of manual assembly curation and re-scaffolding with nf-core/hic workflow ( Servant and Peltzer, 2019) and Salsa ( Ghurye *et al.,* 2019) corrected 54 missing/misjoins. The changes were visualized with a Hi-C contact map using Juicer ( Durand *et al.,* 2016b). JupiterPlots ( Chu, 2018) was used to perform scaffold-level alignment with Green turtle reference genome and generate synteny plot for synteny analysis. The final sequence was analyzed using BlobToolKit ( Challis *et al.,* 2020) for quality assessment and RepeatMasker ( Tarailo‐Graovac & Chen, 2009) for annotation of repetitive regions. The parameter and version number of software tools are listed in Table 3.

## Results

### Genome sequence report

**Figure 1.**
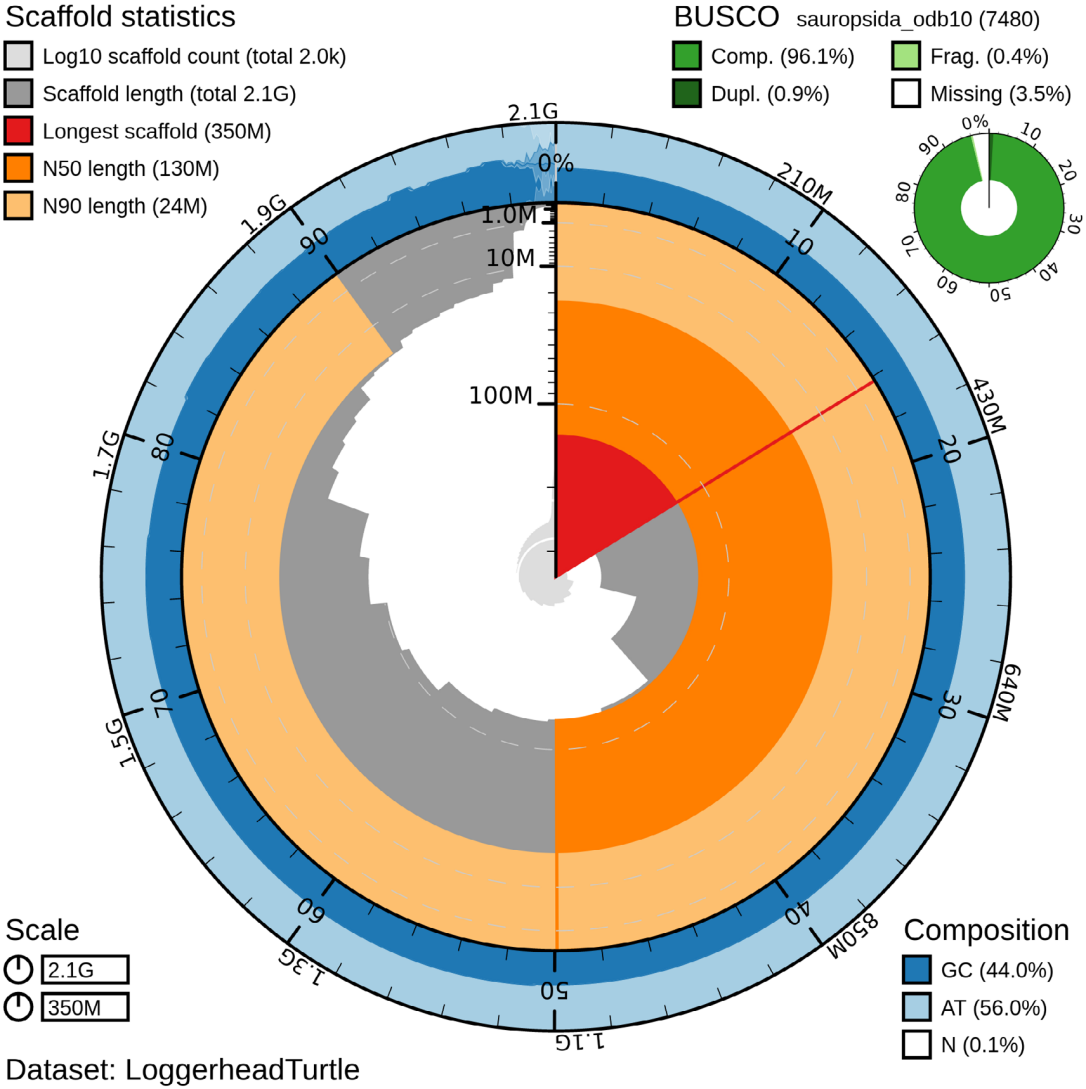
Genome assembly of *Caretta caretta*, rCarCar2: metrics. Snail plot showing N50 metrics, base pair composition and BUSCO gene completeness for *C. caretta* (rCarCar2) generated from Blobtoolkit v.2.6.4 ( Challis *et al.,* 2020). The plot is divided into 1,000 size-ordered bins around the circumference with each bin representing 0.1% of the 2,134,012,717 bp assembly. The distribution of chromosome lengths is shown in dark grey with the plot radius scaled to the longest chromosome present in the assembly (345,741,823 bp) shown in red. Orange and pale-orange arcs show the N50 and N90 chromosome lengths (130,956,235 and 23,648,662 bp, respectively). The pale grey spiral shows the cumulative chromosome count on a log scale with white scale lines showing successive orders of magnitude. The blue and pale-blue area around the outside of the plot displays the distribution of GC (blue), AT (pale blue) and N (white) percentages using the same bins as the inner plot. A summary of complete (96.1%), fragmented (0.4%), duplicated (0.9%), and missing (3.5%) BUSCO genes in the sauropsida_odb10 set is show in the top right.

The genomes of two unrelated loggerhead sea turtles were sequenced from the same population collected from the Fondazione Cetacea hospital, Riccione, Italy. A total of 39-fold coverage in Nanopore PromethION long reads were generated from a single adult female. Approximately 50-fold coverage in Illumina NovaSeq6000 150 bp paired-end (PE150) reads and 18-fold coverage in Illumina NovaSeq6000 Hi-C sequencing were generated from a second individual. Primary assembly contigs from Nanopore data were further polished with Illumina PE150 shotgun sequencing data and scaffolded with Hi-C data. The final assembly has a total length of 2.13 Gb in 2007 sequence scaffolds with a scaffold N50 of 130.95 Mb ( Table 1). The majority (98.0%) of the assembly sequence was assigned to 28 chromosomal-level scaffolds representing the species’ known 28 autosomes ( Kamezaki, 1989, Machado *et al.*, 2020) (numbered by sequence length; Figure 1– Figure 4; Table 2). Aligned reads from the second turtle to the final assembly had an estimated heterozygosity of 0.11% (2,449,606 heterozygous hits). Determining gene coverage using BUSCO, we estimated 96.1% gene completeness using the sauropsida_odb10 reference set ( Manni *et al.,* 2021). The assembly was compared to a previous chromosome-scale assembly of the closely-related green sea turtle, *Chelonia mydas ( Wang et al., 2013)*, which has been reported to hybridize with the loggerhead sea turtle ( James *et al.,* 2004, Vilaça *et al.*, 2012). The loggerhead sea turtle assembly showed strong synteny to the green sea turtle assembly, as shown in Figure 5. The primary haplotype (rCheMyd1.pri.v2) of the green sea turtle was downloaded from NCBI on July 16, 2022. The proportions of SINEs, LINEs, LTR elements, and DNA transposons within the genomic sequences were determined to be 1.55%, 8.75%, 0.13%, and 1.10%, respectively.

**Table 1.** Genome data for *Caretta caretta*, rCarCar2.

Project accession data	
Assembly identifier	rCarCar2
Species	*Caretta caretta*
Specimen	SJ_126, SJ_184
NCBI Taxonomy ID	8467
BioProject	PRJNA826225
BioSample ID	SAMN28968396, SAMN27958248
Isolate Information	SJ_184/204:Loco2, SJ_126:Eziel1
**Raw data accessions**	
Oxford Nanopore PromethION	SRX15677840, SRX15677841
Hi-C Illumina	SRX15677843
Illumina short-read	SRX15677842
**Genome assembly**	
Assembly accession	GCA_023653815.1
Assembly name	GSC_CCare_1.0
Span (Mb)	2,134
Number of contigs	2,753
Contig N50 length (Mb)	18,214
Number of scaffolds	2,008
Scaffold N50 length (Mb)	130,956
Longest scaffold (Mb)	345.7
BUSCO * genome score	C:96.1%[S:95.2%,D:0.9%],F:0.4%,M:3.5%,n:7480

* BUSCO scores based on the sauropsida_odb10 BUSCO set using v5.0.0. C=complete [S=single copy, D=duplicated], F=fragmented, M=missing, n=number of orthologues in comparison.

**Figure 2.**
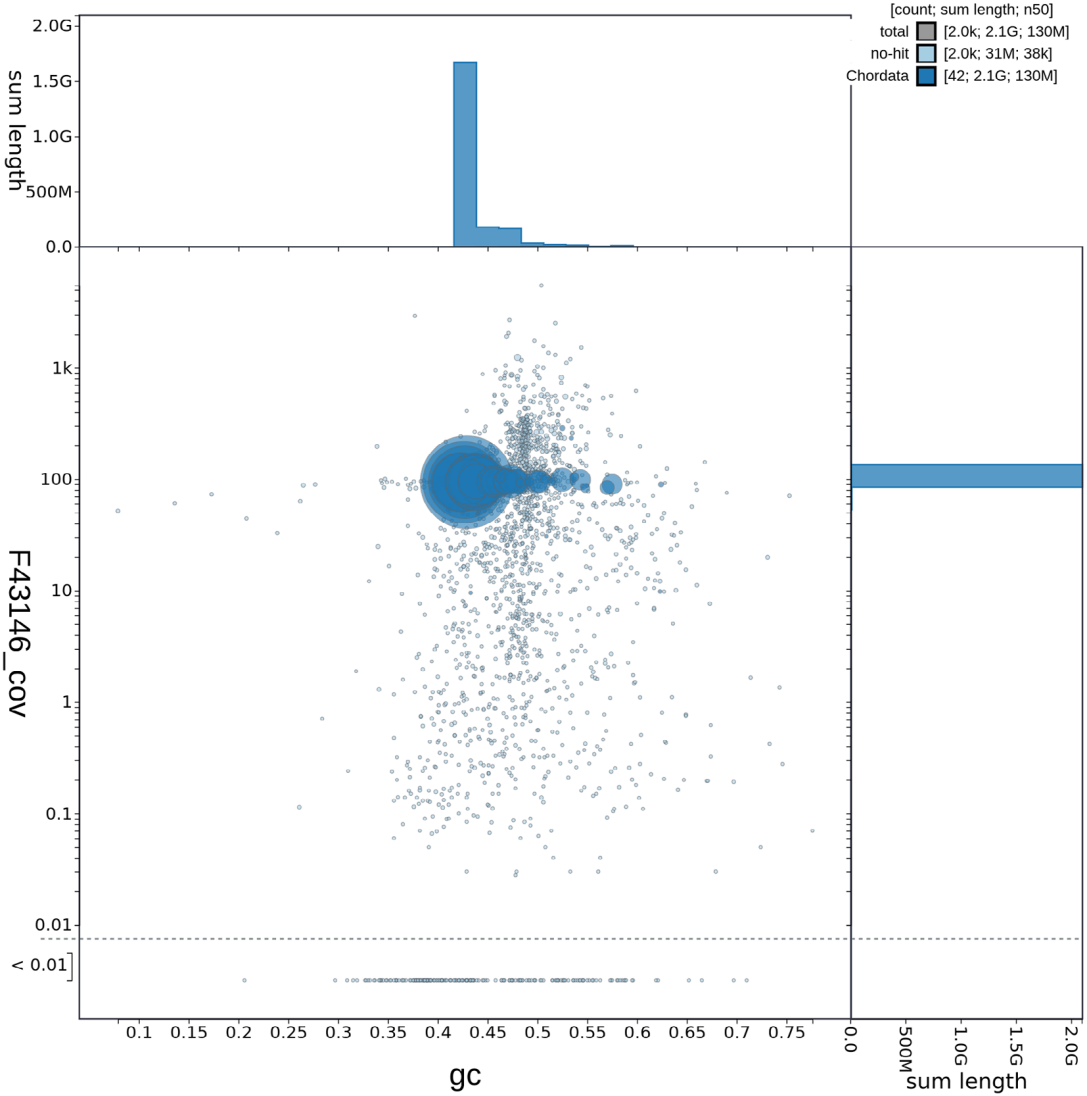
Genome assembly of *Caretta caretta*, rCarCar2: GC-content. GC-coverage plot of *C. caretta* (rCarCar2) generated from Blobtoolkit v.2.6.4 ( Challis *et al.,* 2020). Scaffolds are coloured by phylum with Chordata represented by blue and no-hit represented by pale blue. Circles are sized in proportion to scaffold length. Histograms show the distribution of scaffold length sum along each axis.

**Figure 3.**
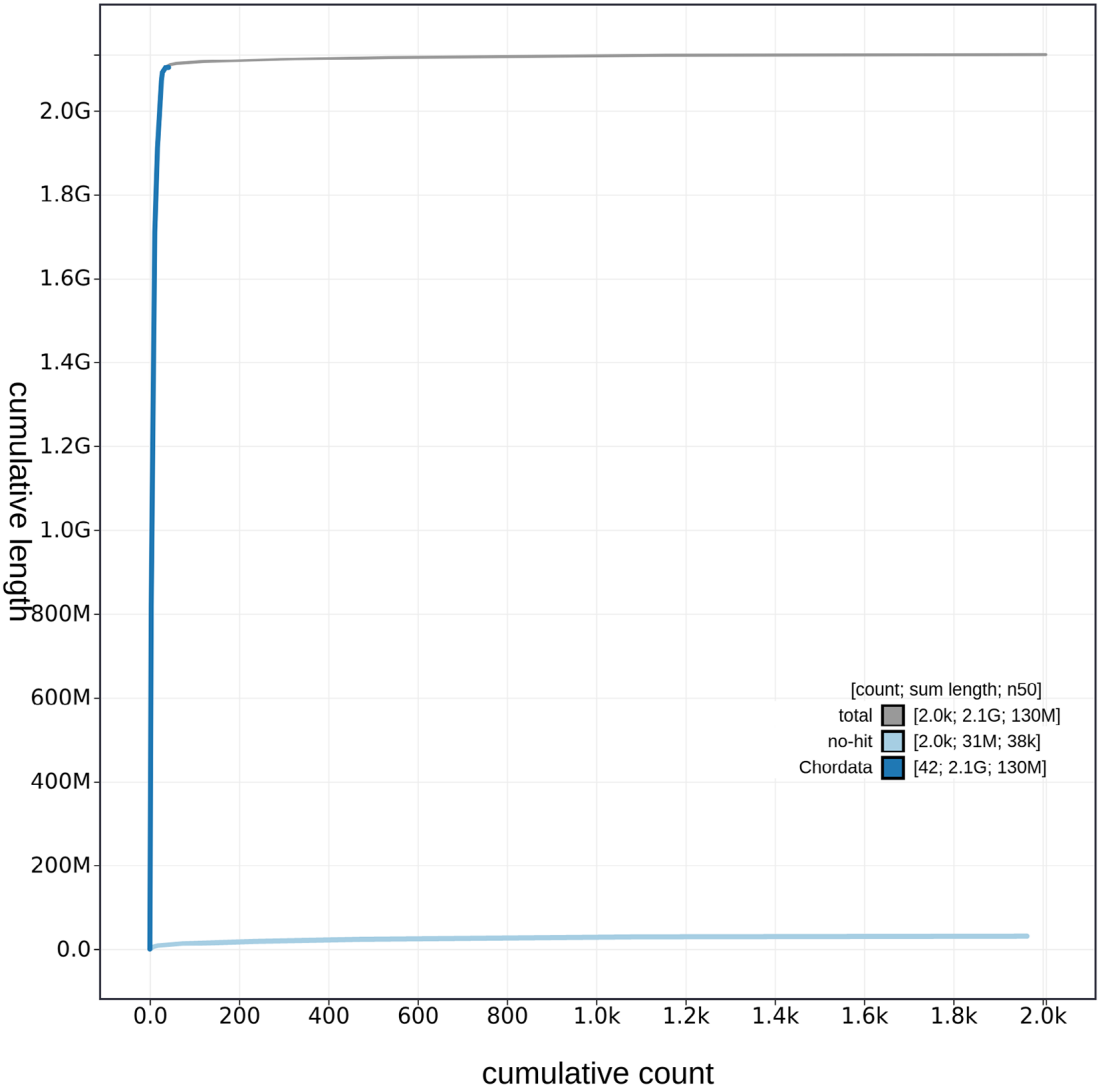
Genome assembly of *Caretta caretta*, rCarCar2: cumulative sequence length. Cumulative sequence length of *C. caretta* (rCarCar2) generated from Blobtoolkit v.2.6.4 ( Challis *et al.,* 2020). The grey line shows the cumulative length for all scaffolds. Coloured lines show cumulative lengths of scaffolds assigned to each phylum using the BUSCO genes tax rule, with Chordata represented by blue and no-hit represented by pale blue.

**Figure 4.**
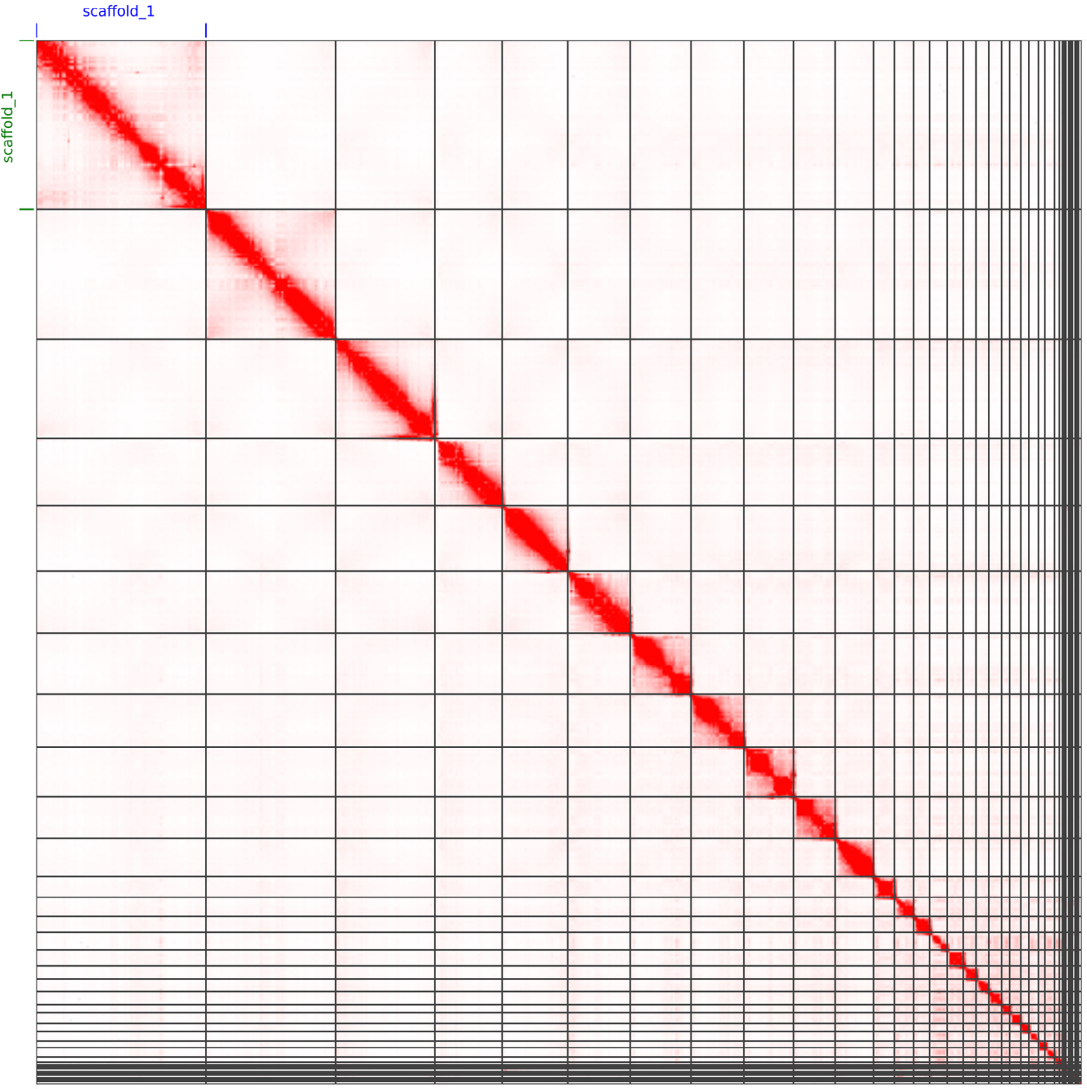
Genome assembly of *Caretta caretta*, rCarCar2: Hi-C contact map. HiC contact map of rCarCar2 assembly visualized using JuiceBox v2.13.07 ( Durand *et al.,* 2016a). Chromosomes are shown in order of size from left to right and top to bottom. As an additional confirmation for the quality of the assembly, the microchromosomes are visible as a cluster of spatially-associated contigs in the lower right, as reported in by Waters *et al.,* 2021.

**Table 2.** Chromosomal pseudomolecules in the genome assembly of *Caretta caretta*, rCarCar2.

RefSeq sequence	Chromosome	Size (Mb)	GC%
NC_064473.1	1	345.74	42.86
NC_064474.1	2	265.32	42.62
NC_064475.1	3	208.08	42.71
NC_064476.1	4	135.63	42.34
NC_064477.1	5	130.96	42.42
NC_064478.1	6	128.66	43.74
NC_064479.1	7	123.31	43.74
NC_064480.1	8	108.54	43.66
NC_064481.1	9	101.34	43.68
NC_064482.1	10	85.28	44.40
NC_064483.1	11	76.53	43.00
NC_064484.1	12	43.19	43.81
NC_064485.1	13	38.20	47.24
NC_064486.1	14	35.79	45.97
NC_064487.1	15	33.48	45.53
NC_064488.1	16	25.69	46.28
NC_064489.1	17	24.70	45.64
NC_064490.1	18	23.65	46.93
NC_064491.1	19	20.21	48.10
NC_064492.1	20	19.04	47.85
NC_064493.1	21	18.99	46.81
NC_064494.1	22	17.93	52.48
NC_064495.1	23	16.78	47.24
NC_064496.1	24	16.65	49.92
NC_064497.1	25	16.37	50.20
NC_064498.1	26	13.31	54.27
NC_064499.1	27	12.55	57.47
NC_064500.1	28	5.34	57.00

**Table 3.** Software tools used.

Software	Version	Parameters	Source
Racon	1.4.13	Default parameters	Vaser *et al.,* 2017
Medaka	1.2.0	Default parameters	https://github.com/nanoporetech/medaka
Pilon	1.23	Default parameters	Walker *et al.,* 2014
Salsa	2.3	-m CLEAN -e GATC,GANTC,CTNAG,TTAA	Ghurye *et al.,* 2019
BlobToolKit	2.6.4 (BTK pipeline) 3.1.0 (Blobtoolkit)	Default parameters	Challis *et al.,* 2020
nf-core/hic	1.1.0	--restriction_site ‘^GATC,G^ANTC,C^TNAG,T^TAA’ --ligation_site ‘GATCGATC,GANTGATC,GANTANTC,GATCANTC’ --skip_tads	Servant and Peltzer, 2019
Juicer Tools	2.13.06	Default parameters	Durand *et al.,* 2016b
Juice Box	2.13.06	Default parameters	Durand *et al.,* 2016a
Redbean	2.5	Default parameters	Ruan and Li, 2019
LongStitch	1.0.1	tigmint-ntLink-arks G=2e9 z=100	Coombe *et al.,* 2021
Jupiter Plot	1.0	ng=98	Chu, 2018
Busco	5.2.2	-l sauropsida_odb10	Manni *et al.,* 2021
Quast	5.0.2	Default parameters	Gurevich *et al.,* 2013
RepeatMasker	4.1.5	-species “Caretta caretta”	Tarailo‐Graovac & Chen, 2009

**Figure 5.**
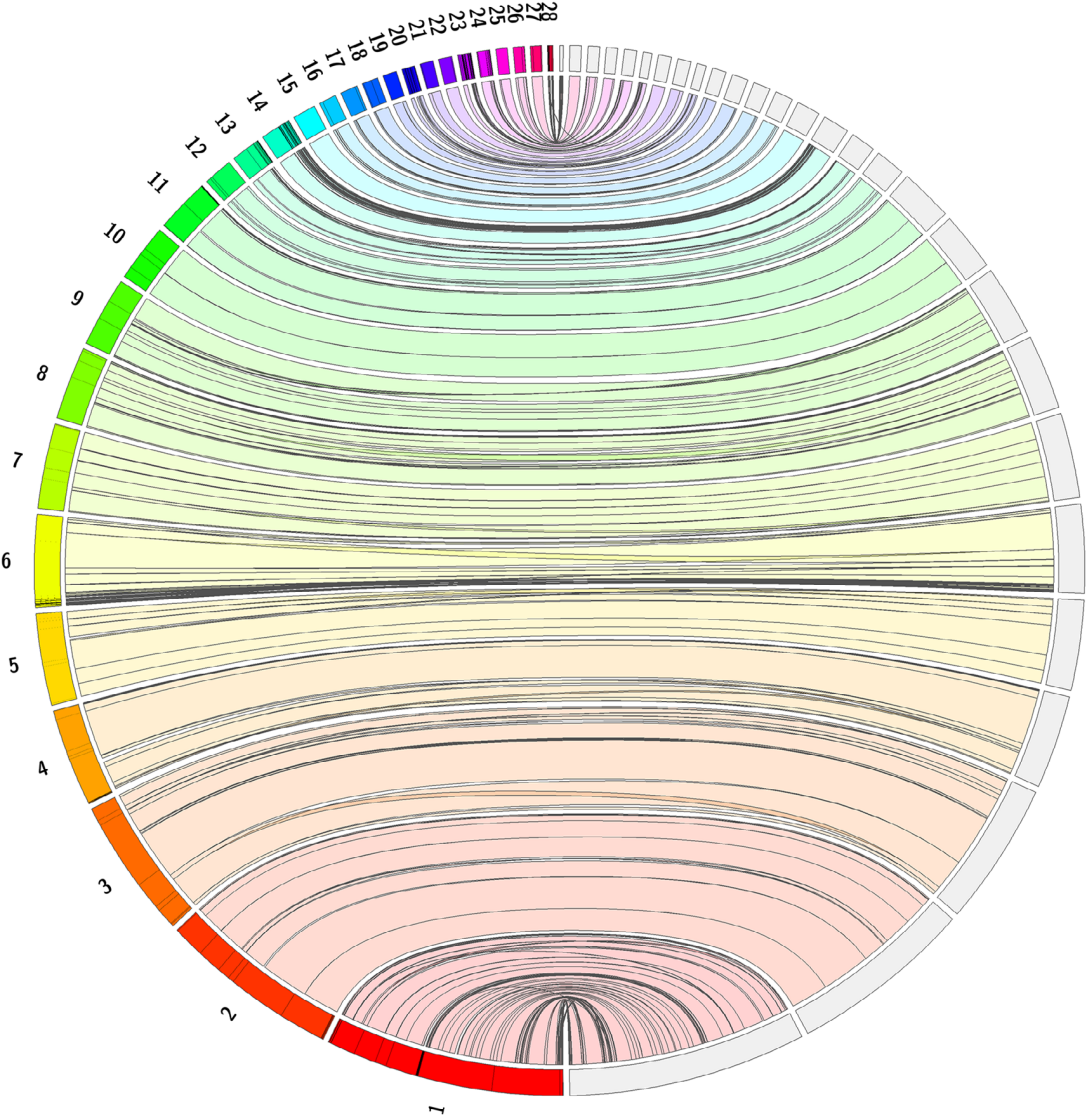
Jupiter plot alignment of *Caretta caretta *with *Chelonia mydas* (green sea turtle). Full genome alignment of *Caretta caretta* genome, rCarCar2 (right), and *Chelonia mydas* (green sea turtle) genome (primary haplotype v2), rCheMyd1 (left), generated using Jupiter Plot ( Chu, 2018). The left of the circle shows 28 green sea turtle chromosomes and the right of the circle shows 28 loggerhead sea turtle chromosomes. Coloured bands represent synteny between the genomes, and lines crossing the circle indicate genomic rearrangements, or break points in the scaffolds.

### Genome annotation

The loggerhead sea turtle genome assembly was annotated by both RefSeq annotation pipeline ( Li *et al.*, 2020) and Ensembl gene annotation system ( Aken *et al.*, 2016). The RefSeq annotation pipeline includes 24,923 genes and pseudogenes, and 54,583 mRNA transcripts ( NCBI Caretta caretta Annotation Release). The Ensembl annotation includes 19,633 coding genes, 4,161 non-coding genes and 42,302 mRNA transcripts ( Caretta caretta - Ensembl Rapid Release).

## Data Availability

National Centre for Biotechnology Information BioProject: Loggerhead Sea turtle ( *Caretta caretta*) genome sequencing and assembly, rCarCar2. Accession number: PRJNA826225. The genome sequence is released openly for reuse. The *C. caretta* genome sequencing initiative is part of the Canadian BioGenome Project and CanSeq150 Projects initiatives. All raw sequence data and the assembly have been deposited in INSDC databases. The genome is annotated through the Reference Sequence (RefSeq) database in BioProject accession number PRJNA853764. Raw data and assembly accession identifiers are reported in Table 1.
