# Supplementary material for: The genome sequence of the Loggerhead sea turtle, Caretta caretta Linnaeus 1758
Source: F1000Res. 2023 Jun 27;12:336. Originally published 2023 Mar 27. [Version 2] doi: 10.12688/f1000research.131283.2 (PMC10338980; doi:10.12688/f1000research.131283.2)
Supplement: Peer review history

# Peer review: Reviewer response for version 2

**DOI:** 10.5256/f1000research.151837.r181940

**Published:** 2023-07-12

**Authors:** Cinta Pegueroles

**Affiliations:** 1 Department of Genetics, Microbiology and Statistics, and Institute for Research on Biodiversity (IRBio), Universitat de Barcelona, Barcelona, Catalonia, Spain

I thank the authors for their thorough revisions, which carefully addressed the comments raised. Congratulations for generating this high quality genome of *Caretta caretta. *It is an importance resource for the scientific community and the management of this vulnerable species.

Are the datasets clearly presented in a usable and accessible format, and the assembly and annotation available in an appropriate subject-specific repository?

Yes

Are sufficient details of the sequencing and extraction, software used, and materials provided to allow replication by others?

No

Are the rationale for sequencing the genome and the species significance clearly described?

Yes

Are the protocols appropriate and is the work technically sound?

Yes

Reviewer Expertise:

Genomics, bioinformatics

I confirm that I have read this submission and believe that I have an appropriate level of expertise to confirm that it is of an acceptable scientific standard.

# Peer review: Reviewer response for version 1

**DOI:** 10.5256/f1000research.144107.r168077

**Published:** 2023-04-27

**Authors:** Cinta Pegueroles

**Affiliations:** 1 Department of Genetics, Microbiology and Statistics, and Institute for Research on Biodiversity (IRBio), Universitat de Barcelona, Barcelona, Catalonia, Spain

This manuscript describes the sequencing and annotation of the *Caretta caretta* genome, which is already available in public data bases. It is a high quality genome that for sure is positively impacting the sea turtles community.

 The analyses are appropriate and results are sound (despite I miss more details, see below). A high percentage of contigs were assembled into chromosomes, and assembled chromosomes overall showed conserved synteny with the green turtle.

 I found surprising that there is no information about repetitive elements. Where they annotated? I strongly recommend to report the levels and type of repetitive elements found within the genome. They can be easily annotated using the repeatMasker software.

 In the abstract I recommend to briefly report the quality of the genome assembly, for instance by adding the percentage of complete BUSCO.

 Despite genome notes are short by definition, in general I miss more details of how analyses were performed. For instance, there is no information of the parameters used when running the programs and it is not explained how the syntenic analyses were performed, neither the annotation of the genome.

 Regarding the annotation of the genome, I do not understand this sentence, “The loggerhead sea turtle assembly was also annotated for 54,583 protein sequences using RefSeq (GCF_023653815.1, PRJNA853764)” since there are 19,633 protein coding genes annotated in Ensembl.

 The mitochondrial genome is not reported in this genome note but it is provided in NCBI. I think it should be mentioned here including the tools that were used for its assembly and annotation.

Are the datasets clearly presented in a usable and accessible format, and the assembly and annotation available in an appropriate subject-specific repository?

Yes

Are sufficient details of the sequencing and extraction, software used, and materials provided to allow replication by others?

No

Are the rationale for sequencing the genome and the species significance clearly described?

Yes

Are the protocols appropriate and is the work technically sound?

Yes

Reviewer Expertise:

Genomics, bioinformatics

I confirm that I have read this submission and believe that I have an appropriate level of expertise to confirm that it is of an acceptable scientific standard, however I have significant reservations, as outlined above.

# Response

**Published:** 2023-06-20

**Authors:** Glenn Chang

**Affiliations:** Canada's Michael Smith Genome Sciences Centre, Canada

Dear Dr. Cinta Pegueroles,

 Thank you for your thorough review of our paper. We have carefully considered your comments and have made the following changes to the revised version of the genome note:

1. **Repetitive elements**: We have now used RepeatMaster to annotate the repetitive elements within the genome. The revised paper reports that we found 1.55% SINEs, 8.75% LINEs, 0.13% LTR elements, and 1.10% DNA transposons within the genomic sequences, as mentioned in the Results section.
2. **Abstract QC metrics**: We have included the busco score and N50 in the abstract to provide quality control metrics right from the beginning.
3. **Software Parameters**: Table 3 now includes the parameters used for each software involved in the genome assembly. In addition to the software name, version, and source, we have added a new column specifically stating the parameters used.
4. **Syntenic analyses**: We have made it more explicit in the paper that JupyterPlot was specifically used to perform scaffold-level alignment and synteny plots for the syntenic analysis.
5. **Gene Annotation pipeline**: The revised paper now clearly states that this genome underwent two annotation pipelines, namely the RefSeq annotation pipeline and the Ensembl gene annotation system. We have provided clearer results for both annotations in the genome annotation section.
6. **Mitochondria**: We did not examine the mitochondrial genome in this study. However, it was automatically grouped with our genome by NCBI. The other mitochondrial genome study can be found here: https://pubmed.ncbi.nlm.nih.gov/22295859/

Thank you once again for your time and valuable feedback during the review process. We believe that these changes have strengthened our paper and addressed your suggestions appropriately.

# Peer review: Reviewer response for version 1

**DOI:** 10.5256/f1000research.144107.r168076

**Published:** 2023-04-06

**Authors:** Richard Challis

**Affiliations:** 1 Tree of Life, Wellcome Sanger Institute, Hinxton, England, UK

Chang et al. present a chromosomal genome assembly of the Loggerhead sea turtle, Caretta caretta, using a combination of Nanopore long reads, HiC and Illumina. The conservation importance of having a genome assembly for this globally distributed but vulnerable species is made very clear.

 As the second chromosomal assembly of a marine turtle it is informative to see a synteny plot comparing this to the green sea turtle, Chelonia midas. This highlights the strongly conserved synteny, similarity in overall assembly span and relative chromosome sizes between these species while maintaining the concise focussed approach typical of a Genome Note.

 Overall the article was very clearly presented, however the presentation of summary information about the 2 sets of gene annotation was slightly inconsistent and I found myself referring to the RefSeq annotation page to compare the numbers of coding vs no-coding genes with the values presented for the Ensembl annotation.

Are the datasets clearly presented in a usable and accessible format, and the assembly and annotation available in an appropriate subject-specific repository?

Yes

Are sufficient details of the sequencing and extraction, software used, and materials provided to allow replication by others?

Yes

Are the rationale for sequencing the genome and the species significance clearly described?

Yes

Are the protocols appropriate and is the work technically sound?

Yes

Reviewer Expertise:

Genomics, Bioinformatics

I confirm that I have read this submission and believe that I have an appropriate level of expertise to confirm that it is of an acceptable scientific standard.

# Response

**Published:** 2023-06-20

**Authors:** Glenn Chang

**Affiliations:** Canada's Michael Smith Genome Sciences Centre, Canada

Dear Dr. Richard Challis,

 Thank you for reviewing our genome note and providing valuable comments. We have carefully considered your comments and made the necessary revisions to address your concerns.

 In particular, we have taken steps to clarify the genome annotation sections. We have made the results of the RefSeq and Ensembl annotation pipelines more distinct in the paper. Additionally, we have provided hyperlinks to both sets of results, allowing readers to access them directly.

 Once again, we sincerely appreciate your time and effort in reviewing our genome note. We believe that the changes we have made effectively address your concerns and improve the clarity of our paper.
